# Current knowledge on radon risk: implications for practical radiation protection? radon workshop, 1/2 December 2015, Bonn, BMUB (Bundesministerium für Umwelt, Naturschutz, Bau und Reaktorsicherheit; Federal Ministry for the Environment, Nature Conservation, Building and Nuclear Safety)

**DOI:** 10.1007/s00411-016-0657-2

**Published:** 2016-06-22

**Authors:** Wolfgang-Ulrich Müller, Augusto Giussani, Werner Rühm, Jean-Francois Lecomte, John Harrison, Michaela Kreuzer, Christina Sobotzki, Joachim Breckow

**Affiliations:** Institut für Medizinische Strahlenbiologie, Universitätsklinikum Essen, 45122 Essen, Germany; Federal Office for Radiation Protection, Ingolstädter Landstr. 1, 85764 Oberschleissheim, Germany; Institute of Radiation Protection, Helmholtz Zentrum München, German Research Center for Environmental Health, Ingolstädter Landstr. 1, 85764 Neuherberg, Germany; Institut de Radioprotection et de Sûreté Nucléaire, International Affaires Directorate, P.O. Box 17, 92262 Fontenay-aux-Roses, France; Faculty of Health and Life Sciences, Oxford Brookes University, Oxford, OX3 0BP UK; Institute of Medical Physics and Radiation Protection, THM University of Applied Sciences, 35390 Giessen, Germany

**Keywords:** Radon, Dose conversion factor, Lung cancer

## Abstract

ICRP suggested a strategy based on the distinction between a protection approach for dwellings and one for workplaces in the previous recommendations on radon. Now, the Commission recommends an integrated approach for the protection against radon exposure in all buildings irrespective of their purpose and the status of their occupants. The strategy of protection in buildings, implemented through a national action plan, is based on the application of the optimisation principle below a derived reference level in concentration (maximum 300 Bq m^−3^). A problem, however, arises that due to new epidemiological findings and application of dosimetric models, ICRP 115 (Ann ICRP 40, 2010) presents nominal probability coefficients for radon exposure that are approximately by a factor of 2 larger than in the former recommendations of ICRP 65 (Ann ICRP 23, 1993). On the basis of the so-called epidemiological approach and the dosimetric approach, the doubling of risk per unit exposure is represented by a doubling of the dose coefficients, while the risk coefficient of ICRP 103 (2007) remains unchanged. Thus, an identical given radon exposure situation with the new dose coefficients would result in a doubling of dose compared with the former values. This is of serious conceptual implications. A possible solution of this problem was presented during the workshop.

## Background

In its publication 65, the International Commission on Radiological Protection (ICRP) suggested dose conversion factors for radon of 5 mSv per working level month (WLM) for workplace exposures and 4 mSv WLM^−1^ for domestic exposures. These values were based on the so-called epidemiological approach, comparing the total detriment as defined in ICRP 60 (1991) (5.6 × 10^−2^ Sv^−1^ for workers, 7.3 × 10^−2^ Sv^−1^ for public) with the lung cancer risks for miners of 2.83·10^−4^/WLM. ICRP 115 (2010) updated the lung cancer risk to 5.0 × 10^−4^ WLM^−1^, which, together with the new values of the total detriment as defined in ICRP 103 (2007) (4.2 × 10^−2^ Sv^−1^ for workers, 5.7 × 10^−2^ Sv^−1^ for public), provided new estimates for the dose conversion factors: 12 mSv WLM^−1^ for workplace exposures and 9 mSv WLM^−1^ for domestic exposures.

As a result, there is about a factor of 2 difference between the estimates of ICRP 65 (1993) and those of ICRP 115 (2010), both obtained with the epidemiological approach. It should be noted that the epidemiological dose conversion approach compares the detriment for two different situations: the detriment per Sv based on incidence of total cancers and hereditary effects evaluated in a Japanese population after an acute external exposure to low-LET radiation is compared with the detriment per WLM in terms of lung cancer mortality evaluated in a male adult population (miners) after a prolonged internal exposure to high-LET radiation from incorporated alpha emitters.

ICRP 105 (2010) has recommended that radon dose coefficients should in future be calculated using biokinetic and dosimetric models (dosimetric approach), as done for all other radiologically relevant radionuclides. Biokinetic models are used to describe the deposition and distribution of activity in the human body and dosimetric models to calculate equivalent doses to the target organs. With this approach, effective dose coefficients of 14 mSv WLM^−1^ for domestic exposures and between 12 and 21 mSv WLM^−1^ for workplace exposures (depending on type of activity) were estimated.

In an effort to discuss the differences in dose conversion coefficients mentioned above, the German Commission on Radiological Protection (Strahlenschutzkommission SSK) organised a workshop on this issue, to foster the current discussion on radon risk.

A Böttger (Bundesministeriums für Umwelt, Naturschutz, Bau und Reaktorsicherheit BMUB, Federal Ministry for the Environment, Natur Conservation, Building and Nuclear Safety) gave a welcome address to the foreign speakers and also to those from BMUB, BfS (Bundesamt für Strahlenschutz BfS, Federal Office for Radiation Protection) and SSK. He welcomed also F Shannoun from UNSCEAR (United Nations Scientific Committee on the Effects of Atomic Radiation) and C Streffer a long-standing member from ICRP, as well as representatives from the German states. He said that ICRP works to maintain the system of radiological protection, to broaden the awareness of radiation protection and to allow access to ICRP recommendation. He brought to mind the Darby study from 2005 (Darby et al. 2005) which gave an additional risk of lung cancer after indoor radon exposure of 0.16 per 100 Bq m^−3^. For Germany, natural sources of ionising radiation contribute to about 2 mSv annual effective dose, in addition to a similar value from diagnostic X-ray procedures. However, with the new coefficients proposed by ICRP, the effective dose from radon would be doubled. He raised a number of questions: Is the concept of radiological protection still consistent? Is there solid evidence for the change, and is there still trust in the existing system? Was a change necessary at all? Are there consequences for radiological protection? Are medical exposures now less important? Finally, he expressed the concern that it will be too late to include the new recommendations into the upcoming implementation of the Basic Safety Standards.

In the following, first, the four presentations are summarised. Subsequently, the essential issues of the overall discussion are described and an overall conclusion is drawn.

## ICRP126: radiological protection against radon exposure (J-F Lecomte)

In 2009, the Main Commission of the ICRP approved the development by the Committee 4 of updated guidance on radiological protection against radon exposure. The draft report has been posted on the ICRP website for public consultation in 2012. It has been approved by the Main Commission in early 2014 and published as publication 126 in late 2014. The objective of the new publication is to describe and clarify the application of the Commission’s system to the protection of members of the public and workers against radon exposures in dwellings, workplace and other types of locations. ICRP 126 (2014) is considering the recently consolidated ICRP general recommendations, the new scientific knowledge about the radon risk and the experience gained by many organisations and countries in the control of radon exposure. It is mainly focused on radon 222.

In summary, in the previous recommendations on the same subject [ICRP 65 (1993); ICRP 103 (2007)], the Commission recommended a strategy based on the distinction between a protection approach for dwellings and a protection approach for workplaces. Now, the Commission recommends an integrated approach for the protection against radon exposure in all buildings in which radon exposure occurs whatever the purpose of the building and the types of its occupants (member of the public, worker, smoker, non-smoker, etc.). The strategy of protection in buildings, implemented through a national action plan, is based on the application of the optimisation principle below a derived reference level in concentration, chosen as low as reasonably achievable (maximum 300 Bq m^−3^) on the basis of a reference level in effective dose of the order of 10 mSv per year. A graded approach is recommended, according to the degree of responsibilities, notably in workplaces, and the priorities determined at national level. The Commission expects a real improvement in the reduction in exposure due to radon, which is by far the main source of public exposure worldwide.

(For additional information, see Lecomte 2014).

## ICRP reasoning and position on changes to nominal risk coefficients and dose coefficients for inhaled radon-222 and progeny (J Harrison)

ICRP 65 (1993) provided an epidemiologically based dose conversion convention, with a value of 5 mSv WLM^−1^ for adults, and 4 mSv WLM^−1^ for all ages. In ICRP 115 (2010), more recent epidemiological data were reviewed, focusing on low levels of exposure and exposure rates in mines, and a revision was proposed of the detriment-adjusted nominal risk coefficient for a mixed adult population of smokers and non-smokers from 2.8 × 10^−4^ to 5 × 10^−4^ WLM^−1^. Comparisons of lung cancer risks for residential exposures with estimates derived for miners showed good agreement. The accompanying Statement on Radon from the Porto meeting [ICRP 115 (2010)] adopted the revised nominal risk coefficient and accordingly changed the recommended reference level from 600 to 300 Bq m^−3^ for dwellings. The statement further indicated the Commission’s intention to apply the same approach to intakes of radon and its progeny as for other radionuclides and to provide dosimetrically based coefficients to replace the dose conversion convention.

Using the revised nominal risk coefficient of 5 × 10^−4^ WLM^−1^, and the ICRP 103 (2007) detriment values, dose conversion convention values of 12 mSv WLM^−1^ for adults and 9 mSv WLM^−1^ for all ages would be derived. The value of 9 mSv WLM^−1^ is obtained without any information on risk of exposure during childhood from radon and progeny. Dose coefficients for inhalation and ingestion of radioisotopes of radon, including inhalation of radon-222 and its radioactive progeny, will be included in Part 3 of a series of reports currently in preparation by ICRP on Occupational Intakes of Radionuclides (OIR series). These data will include values for inhaled radon-222 and progeny of about 11 mSv effective dose WLM^−1^ for exposures in mines and 20 mSv WLM^−1^ for indoor workplaces. However, using a more realistic breathing rate for sedentary occupations such as office workers gives a value of about 14 mSv WLM^−1^ (Harrison and Marsh 2012; Marsh et al. 2010). For dwellings, the dose coefficient was calculated to be about 13 mSv WLM^−1^.

The present situation is a remarkable consistency between coefficients obtained by dosimetric calculations and conversion coefficients based on epidemiological comparisons. Noting that inhaled radon-222 and progeny is a special case for which there is good epidemiology as well as dosimetry, and taking account of the two methods of calculation of dose coefficients, the Commission will recommend the use of a single-dose conversion coefficient of 12 mSv WLM^−1^, equivalent to 3.4 mSv per mJ h m^−3^, for the calculation of doses following inhalation of radon and radon progeny in workplaces. This reference dose coefficient is considered to be applicable to the majority of circumstances with no adjustment for aerosol characteristics. However, in cases where aerosol characteristics are significantly different from typical conditions, where sufficient, reliable aerosol data are available and estimated doses warrant more detailed consideration, it will be possible to calculate site-specific dose coefficients using the biokinetic and dosimetric data to be provided in OIR Part 3 and the accompanying electronic annexes.

In terms of measurements of radon-222 gas exposure, the reference effective dose coefficient of 12 mSv per WLM (3.4 mSv per mJ h m^−3^) corresponds to 7.5 × 10^−6^ mSv per Bq h m^−3^, assuming an equilibrium factor, *F* of 0.4 between radon and its short-lived progeny (Harrison and Marsh 2012). With an occupancy of 2000 h per year for a worker [ICRP 65 (1993); ICRP 115 (2010)] and *F* = 0.4, the effective dose corresponding to annual exposure at the upper references level of 300 Bq m^−3^ recommended in ICRP 126 (2014) is 4.5 mSv. For the reference residential occupancy of 7000 h per year, the corresponding value of effective dose is 15.8 mSv.

Concern has been expressed that increases in radon dose coefficients from ICRP 65 (1993) values by a factor of two or more for domestic exposures will result in changes in estimates of the contribution of radon to annual average exposures of the population in different countries and hence to the relative proportions contributed by medical and natural environmental sources. While this is a legitimate and important communication issue, this use of effective dose goes beyond its intended use within the system of protection. Nevertheless, effective dose can be seen as a useful tool for the communication of information on inferred risks related to doses from different sources, if the associated uncertainties are appropriately recognised. For inhaled radon-222 and progeny, an important factor demonstrated to have a strong effect on risk is smoking prevalence in the exposed population [ICRP 115 (2010)].

## Radon dose conversion: an epidemiological perspective (M Kreuzer, C Sobotzki)

### Background

The epidemiological “dose conversion convention” is based on a comparison of the detriment per unit of exposure to radon progeny in WLM derived from miner studies with the detriment per unit effective dose in Sv, estimated mainly from the Japanese atomic bomb survivor studies. Based on lifetime excess absolute risk (LEAR) calculations, specific reference background rates and risk models derived from pooled analyses of miner studies, a nominal risk coefficient for lung cancer of 5 × 10^−4^ per WLM was recently recommended by ICRP 115 (2010), which replaces the former value of 2.8 × 10^−4^ per WLM [ICRP 65 (1993)].

To calculate the lifetime risk up to 90 years of age in the miner studies, a constant annual exposure of 2 WLM from 18 to 64 years of age was used as exposure scenario [ICRP 115 (2010)]. The applied projection models were relative risk models, assuming a linear relationship between cumulative radon exposure and lung cancer mortality including effect modifiers for time, age and exposure rate.

### Models considered by ICRP

Regarding radon, the old nominal coefficient was based on the so-called Jacobi or ICRP 65 model applied to data of a pooled analysis of six cohorts including about 30,000 miners and 912 lung cancer deaths (Jacobi et al. 1992; ICRP 65 (1993); Tomasek et al. 2008a). This model took into account the effect modifiers age at exposure and time since exposure, but not exposure rate. The new nominal coefficient was derived from two pooled uranium miner studies—the pooled analyses of 11 miner cohort studies (NRC 1999) and the French/Czech pooled analyses (Tomasek et al. 2008b)—with low radon concentrations, long duration of follow-up and good-quality exposure data.

The pooled analysis of 11 miner cohort studies included about 60,000 miners and 2600 lung cancer deaths and accumulated 1.2 Mio person-years at risk (NRC 1999). The BEIR VI exposure–age–concentration model with three categorical effect modifiers attained age, time since exposure and exposure rate was applied, and the ERR/WLM for exposure rates <0.5 WL was obtained. The French/Czech study included 10,000 miners and 574 lung cancer deaths with 0.25 Mio person-years at risk (Tomasek et al. 2008b). The linear relative risk model included the effect modifiers age at median exposure, time since median exposure, exposure rate and method of exposure assessment (measured/estimated). Exposure rate turned out to be no statistically significant modifier in this low-dose study and was omitted from the final model. Since the method of exposure assessment was a statistically significant modifier, the ERR/WLM for measured exposures was taken.

**Fig. 1:**
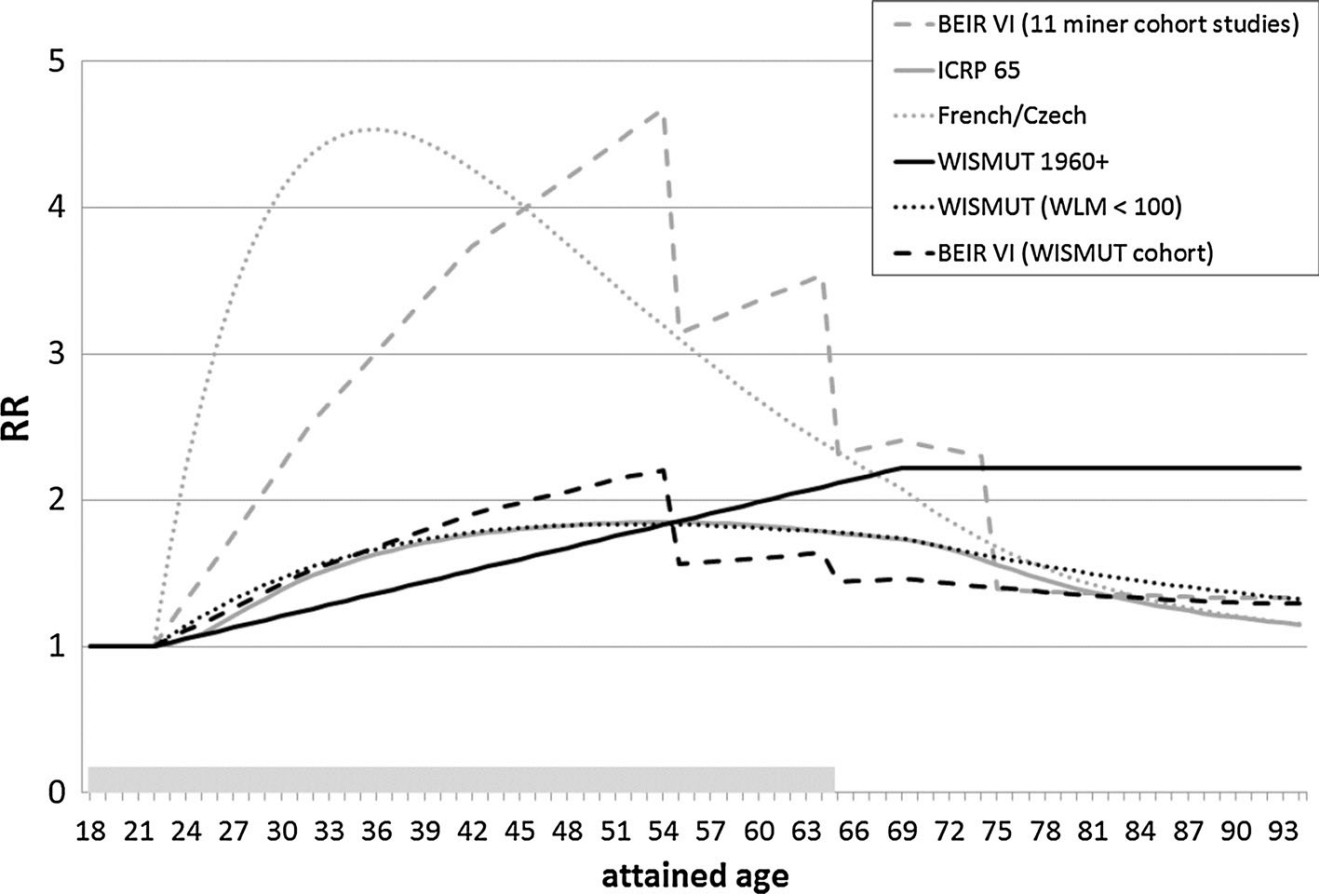
Relative risk (RR) for death from lung cancer in dependence of age for an annual exposure to radon of 2 WLM at ages 18–64 years and a 5-year latency period for different studies. Three pooled studies (NRC 1999; ICRP 1993; Tomasek et al. 2008b) and the German uranium miner cohort study using three different methods to determine the risk at low radon exposures or exposure rates

The calculated LEAR was two times higher for the BEIR VI (11 miner cohort studies) and the French/Czech study compared to the ICRP 65 model (Tomasek et al. 2008a). This is illustrated in Fig. 1, which shows the relative risk (RR) for death from lung cancer in dependence of age for the above-mentioned exposure scenario (annual exposure of 2 WLM at ages 18–64 years and a 5-year latency period) for the three models. Before the age 75 years, the estimated risks in the BEIR VI and French/Czech model are substantially higher than that of the ICRP 65 model, while for ages above 75 years, all risk models predict nearly similar risks. The difference might be explained by the inverse exposure rate effect, which was not considered in the Jacobi model (Tomasek et al. 2008a).

### Risk models from the German uranium miner cohort study

The German uranium miner cohort study was not part of the pooled analyses of the 11 miner studies (NRC 1999), because it was established at a later stage. This cohort has a similar size (*n* = 59,000 miners) and number of lung cancer deaths (*n* = 2900 by end of 2003; *n* = 3500 by end of 2008) as the pooled 11 miner cohort studies together. The number of person-years at risk in the Wismut cohort is two times higher (2.2 Mio) due to more than 60 years of mortality follow-up (1946–2008). The cohort covers a full range of exposures to radon progeny (0–3224 WLM) with a mean of 242 WLM. In order to compare the ERR/WLM for lung cancer at low cumulative radon exposures or exposure rates with those used for lifetime risk calculation by ICRP, the following methods and models were used (see Table 1):

**Table 1:** Description of three different methods to determine the excess relative risk at low radon exposures and exposure rates in the Wismut cohort

	BEIR VI model for exposure rates <0.5 WL	Restriction to person-years at risk <100 WLM	Restriction to workers hired in 1960 or later
Follow-up period	1946–2003	1946–2008	1960–2008
# of workers	58,982	58,982	26,766
# of lung cancer deaths	3000	1016	334
Person-years at risk	2,180,700	1,491,250	846,809
Exposure to radon	Exposure rate < 0.5 WL	Mean: 19 WLM Max: 100 WLM	Mean: 17 WLM Max: 333 WLM
Linear model with effect modification by			
Time since exposure	Yes	Yes	No
Age at exposure/attained age	Yes	No	No
Exposure rate	Yes	No	No

*WLM* working level months

- The BEIR VI exposure–age–concentration model as published by Walsh et al. (2010) for exposure rates <0.5 WL. This model is similar to that applied to the pooled analyses of 11 miner cohorts.
- The linear excess relative risk model with exponential effect modifiers time since median exposure, exposure rate and age at median exposure as published in Walsh et al. (2010) with extension to follow-up by end of 2008 and restriction of person-years at risk to less than 100 WLM (not published). The final model included only time since median exposure as statistically significant effect modifier.
- In a separate analysis, the Wismut cohort was restricted to workers hired after 1959 as published in Kreuzer et al. (2015). In this 1960+ subcohort, the radon exposure rate was uniformly low across the years, all radon values were based on extensive ambient measurements, and information on smoking—a potential confounder for the radon-related lung cancer risk—was available for 56 % of the cohort members. None of the potential effect modifiers such as attained age, time since exposure or exposure rate was statistically significant. Therefore, the final model included no effect modifiers. Adjustment of the ERR/WLM for smoking status did not change the risk estimate. Smoking can thus be considered as no important confounder.

As illustrated in Fig. 1, all three methods led to substantially lower RRs compared to the results of the BEIR VI (11 miner cohort studies) and French/Czech study, at least for ages below 70 years. For ages above 70 years, the RRs of the BEIR IV and the “Wismut <100 WLM” model were similar to that from the pooled studies. Due to the young age of the Wismut 1960+ subcohort, no reliable estimates are possible at higher ages, and therefore, no risks are presented.

### Overall evaluation of LEARs

Currently, no LEARs have been calculated for the Wismut cohort. However, Fig. 1 indicates that LEARs derived from the Wismut study would be lower than those from the BEIR VI (11 miner cohort studies) and Czech/French study by a factor of about 2. Generally, the LEARs are highly sensitive to the risk model (ERR/WLM and three modifying factors) and the considered lifetime range in years (Tirmarche et al. 2012). The estimation of effect modification by time and age of the ERR/WLM is a challenge in studies restricted to low exposures or exposure rates due to low statistical power. Such studies usually result in a simple linear model without effect modifiers (see e. g. Figure 2 in Kreuzer et al. 2015). Without taking into account time since exposure, the RR at older ages may be overestimated.

The LEARs also depend on the reference rates used (Tirmarche et al. 2012) and on assumptions of the type of interaction of smoking with radon (multiplicative or sub-multiplicative). Hunter et al. (2015) recently calculated the lifetime risk of radon-induced lung cancer deaths up to age 75 separately for continuing smokers, ex-smokers and never smokers for different models assuming a multiplicative interaction. The BEIR VI (11 miner cohort studies) model at exposure rates <0.5 WL showed a two times higher LEAR compared to the results of the model derived from the European residential radon study (Darby et al. 2005) in all categories of smoking. In the latter model, the unit Bq m^−3^ from the residential radon studies had been converted into WLM according to Hunter et al. (2015). The ERR/WLM in the European residential radon study was 0.012 (95 % CI 0.004; 0.023) and thus comparable to the results of the Wismut 1960+ subcohort (ERR/WLM = 0.013; 95 % CI 0.007; 0.021) (see Kreuzer et al. 2015).

### Conclusion

Calculations of the ERR/WLM in the Wismut cohort indicate lower LEARs than currently used by ICRP. However, confidence limits for the ERR/WLM and the corresponding time- and age-related effect modifiers of all considered studies are estimated to be large and will strongly overlap. Given this fact and all the other uncertainties in estimating the LEAR, it is notable that the estimated LEARs from the different miner studies just differ by a factor of about two and are even compatible with those from residential radon studies.

## An alternative approach for the assessment of the radon dose coefficient (J Breckow)

### Introduction

In its recommendation ICRP 115 (2010) and some subsequent publications (Harrison and Marsh 2012; ICRP 126 (2014), ICRP presents nominal probability coefficients for radon exposure which are approximately by a factor of 2 larger than in the former recommendation of ICRP 65 (1993). The new values are well supported by a variety of epidemiological studies (e.g. Darby et al. 2005; Hunter et al. 2013; Kreuzer et al. 2014). In the present paper, the nominal probability coefficients of ICRP 115 (2010) are assumed as valid.

**Fig. 2:**
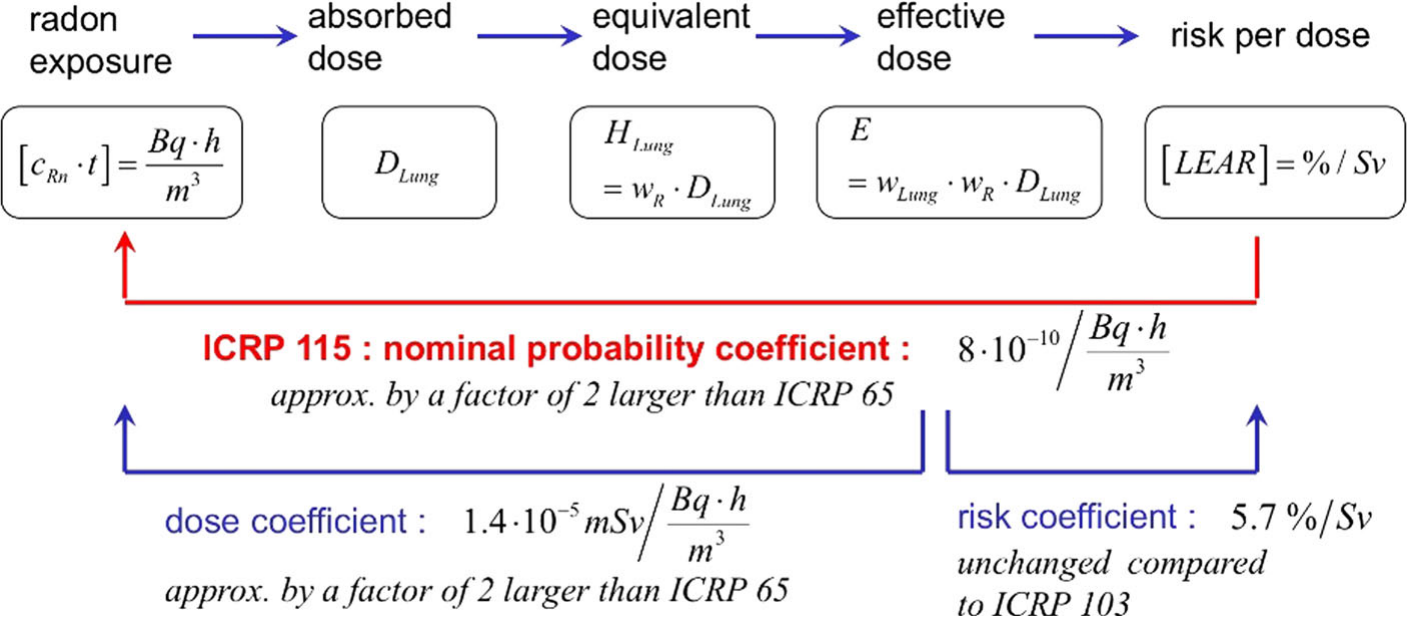
Schematic representation of the series of steps from radon exposure to lifetime excess absolute risk, LEAR. The radon exposure, given in Bq h/m^3^, is the product of radon activity concentration *c*_Rn_ and time *t*. The nominal probability coefficient is for equivalent factor *F* = 1. The dose coefficient of ICRP 115 is approximately by a factor of 2 larger than ICRP 65, whereas the risk coefficient of ICRP 103 is kept unchanged

The nominal probability coefficient indicates the LEAR per effective dose or, respectively, the radiation detriment (both given in % per Sv) due to radon exposure (given either in Bq h m^−3^ or in WLM). The LEAR values in ICRP 115 (2010) are 8 × 10^−10^/Bq h m^−3^ (with equilibrium factor *F* = 1) and 5 × 10^−5^/WLM, respectively. The nominal probability coefficient links the very beginning of a chain of various steps from radon exposure to the very end of the chain, i.e. the corresponding excess risk (Fig. 2). Initially, it does not state explicitly the radon dose.

The factor 2 of increase in ICRP 115 (2010) with respect to ICRP 65 (1993) principally might be situated at any step, or even distributed over several steps, in the sequence of conversions from exposure to risk. ICRP 115 (2010); ICRP 126 (2014); and Harrison and Marsh 2012) set the last step, i.e. the risk coefficient as a conversion from effective dose to detriment (Fig. 2), unchanged with respect to ICRP 103 (2007), while the factor 2 refers to the radon dose coefficient, i.e. the conversion from exposure to effective dose. Thus, with the new ICRP radon dose coefficients, a given radon exposure situation now results in a doubling of dose.

### The epidemiological approach

ICRP 65 (1993) recommended that doses from radon and its progeny should be calculated using a “dose conversion convention” based on epidemiological data (the so-called epidemiological approach). By this approach, the dose is converted into practical action levels set in terms of radon exposure (either in Bq h m^−3^ or in WLM).

The dose conversion convention is a rather simple approach: in order to obtain a dose–exposure relation, the radon probability coefficient, i.e. the lung cancer mortality risk LEAR_L_ per radon exposure (given in WLM^−1^), is put in relation to the risk coefficient, i.e. the total detriment *d*_tot_. Due to the poor prognosis of lung cancer, LEAR_L_ equals approximately to the detriment for lung cancer. For example, with the ICRP 115 (2010) probability coefficient and the ICRP 103 (2007) risk coefficient for the general public, the radon dose coefficient is obtained by:

1 $$\frac{{{{LEAR_{L} } \mathord{\left/ {\vphantom {{LEAR_{L} } {c_{Rn} \cdot t}}} \right. \kern-0pt} {c_{Rn} \cdot t}}}}{{d_{tot} }} = \frac{{{{5 \cdot 10^{ - 4} } \mathord{\left/ {\vphantom {{5 \cdot 10^{ - 4} } {WLM}}} \right. \kern-0pt} {WLM}}}}{{{{5.7 \cdot 10^{ - 2} } \mathord{\left/ {\vphantom {{5.7 \cdot 10^{ - 2} } {Sv}}} \right. \kern-0pt} {Sv}}}} = {{8.8\;mSv} \mathord{\left/ {\vphantom {{8.8\;mSv} {WLM}}} \right. \kern-0pt} {WLM}}$$

with *c*_Rn_: radon activity concentration, *t*: exposure time

A similar calculation for workers in terms of the dose conversion convention result in a dose coefficient of 12 mSv WLM^−1^.

There is a somewhat strange consequence of using the dose conversion convention: The risk of high-LET radiation (radon) is connected to risks of low-LET radiation via the organ-specific detriment. Risk estimates and dose coefficients for the one affect the risk estimates and dose coefficients for the other (because the detriment is equal for both). Thus, whenever risk estimates are revised for other organs than lung and other radiation qualities than radon, this implies alteration for radon as well and vice versa. It is hard to apprehend that the dose due to radon should be dependent on the risk, e.g. due to gamma radiation. Not at least for this striking shortcoming, ICRP 115 (2010) announces its intention to replace the current dose conversion convention with a dosimetric approach (see below). Nevertheless, ICRP emphasises the good agreement between the epidemiological and dosimetric approach. However, in view of the obvious limitations mentioned above, a good agreement by its own may not hold a very strong argument for either.

### The dosimetric approach

ICRP 115 (2010) concludes that radon and its progeny should be treated in the same way as other radionuclides, that is bringing radon into line with all other internal emitters. Thus, radon and its progeny should be calculated using biokinetic and dosimetric models, the so-called dosimetric approach. The dosimetric approach considers a range of parameters relevant to doses from radon, the values for which may change depending on the circumstances of exposure. Thus, any given concentration of radon may result in different doses depending on the circumstances [ICRP 115 (2010)].

Due to a variety of assumptions regarding the anatomy and energy deposition within the lung, biokinetic models provide values for the organ absorbed dose to the lung per unit exposure (given in mGy per WLM). In turn, using the radiation weighting factor for alpha radiation and using the tissue weighting factor for the lung the effective dose per unit exposure can be calculated (given in mSv per WLM). Depending on the exposure scenario, the values of effective dose range from about 10 to 20 mSv per WLM derived using the human respiratory tract model (HRTM) as one of the most relevant biokinetic models [ICRP 66 (1994), ICRP 115 (2010)].

There are several sources of variability and uncertainty including, for example, the activity size distribution of the radon progeny aerosol, the breathing rates, the aerosol deposition in the respiratory tract and others [ICRP 115 (2010)]. Due to these variabilities and uncertainties, it may be estimated that the calculated radon dose coefficients may vary by a factor of about 2 or 3 according to the model parameters considered.

### An alternative approach

**Fig. 3:**
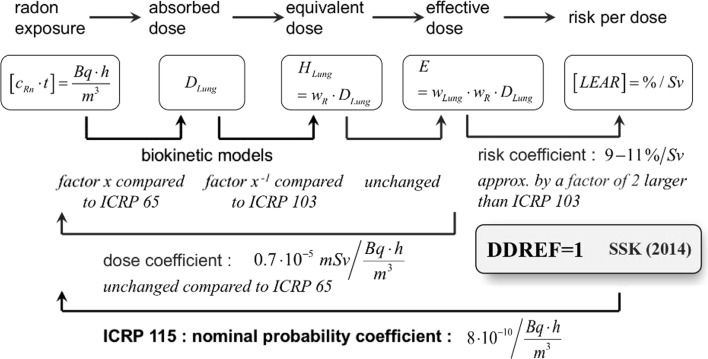
Schematic representation of the series of steps from radon exposure to lifetime excess absolute risk, LEAR. The radon exposure, given in Bq h/m^3^, is the product of radon activity concentration *c*_Rn_ and time *t*. The nominal probability coefficient is for equivalent factor *F* = 1. The lung absorbed dose *D*_Lung_ is determined by biokinetic models. The lung equivalent dose *H*_Lung_ and the effective dose E are obtained by the radiation weighting factor *w*_R_ (changed or unchanged) and the tissue weighting factor *w*_T_, respectively. With the alternative approach, the dose coefficient remains unchanged compared with ICRP 65, whereas the risk coefficient is approximately by factor of 2 larger than ICRP 103

There could be a possibly interesting alternative approach to attenuate some of the limitations with both the epidemiological approach and the dosimetric approach. The (revised) cancer risk per unit radon exposure may be considered as being composed of the dose coefficient for radon and the detriment-adjusted nominal risk coefficient per unit equivalent dose that is identical for all radiation qualities. The doubling (or whatever) of the risk estimates may be represented by doubling the dose coefficient and keeping the risk coefficient unchanged (as proposed by ICRP) or the other way around, by keeping the dose coefficient constant and changing the risk coefficient (as proposed in this paper; Fig. 3).

At first sight, the latter seems to imply major consequences that are of even more tremendous impact to radiation protection: changing risk estimates (e.g. by a factor of 2) are related to all cancer sites and all exposure situations, whereas changing the dose conversion factor relates only to lung and only for radon.

At second sight, however, the situation may be as follows: revisions of risk estimates on account of new scientific findings are generally accepted by both the scientific community and even by the public. In the light of a growing volume of epidemiological studies with increasing resiliency, it is principally not unlikely that a given radiation exposure situation over the years may be seen necessary to be associated with a modified risk. A dose quantity, however, generally is considered to represent an objective or physically firm quantity. It is hardly acceptable that changing risk estimates would result in different dose values for the same exposure situation.

In the past and at present as well, the quantities of risk estimates repeatedly have been under discussion. In its recommendation 103 [ICRP 103 (2007)], ICRP introduced the last comprehensive, extensive and detailed derivation of the quantities and values of risk estimates. By this, it defined the detriment concept to quantify the damage due to radiation exposure. Thus, one of the most important quantities is the so-called nominal risk coefficient that indicates the detriment due to radiation exposure per unit effective dose. For example, for the general public the detriment-adjusted risk coefficient is 5.7 % per Sv.

The assessment of the nominal risk coefficients in ICRP 103 (2007) was performed with a variety of assumptions and modifying parameters. Among others, the so-called dose and dose rate effectiveness factor (DDREF) is of major relevance. The DDREF is a remarkably subtle factor with a considerable conceptual and quantitative influence when it comes to radiation protection. Justification for retaining/modifying/abolishing such a “factor” is therefore not only based on radiobiological or radioepidemiological findings, but also gives rise to questions relating to radiation protection requirements for operational implementation.

For practical radiation protection purposes, it is assumed that stochastic radiation effects are proportional to the dose. This assumption forms what is known as the linear no-threshold (LNT) model, which is one of the basic concepts with major consequences for the entire field of radiation protection. However, radiobiological and radioepidemiological studies indicate deviations from “pure” linearity at low doses and the possibility of dependencies on the dose rate. Such influences would lead to an overestimate of the radiation risk determined on the basis of the LNT model, which is why in previous recommendations, ICRP developed a concept summarising all of these influences into a common factor, namely the DDREF. The nominal risk coefficients for low doses and low dose rates calculated by linear extrapolation are divided by the DDREF. In recommendation ICRP 103 (2007), the ICRP confirms its previous argumentation and recommends retaining a DDREF of 2 for solid tumours in the case of photon and electron exposures (sparsely ionising radiation) [ICRP 103 (2007)].

Already prior to discussions surrounding ICRP 103 (2007), the German Strahlenschutzkommission (SSK, Commission on Radiological Protection) adopted a critical stance to the DDREF concept, not in the least due to its design, and called for the DDREF to be abolished, i.e. a DDREF of 1 (SSK 2006). In a recent recommendation, the SSK (2014) confirmed its previous statements.

In consequence of abolishing the DDREF, the risk coefficients (nota bene: for all organs including lung, and for all radiation qualities including radon) would increase by a certain factor (approximately by a factor of 2). Thus, roughly spoken, the assumed doubling of the radon-induced lung cancer risk per unit exposure may be represented by setting DDREF = 1 and, however, keeping the dose coefficient unchanged with respect to ICRP 65 (1993). This is the central idea of the alternative approach presented here (Fig. 3).

**Fig. 4:**
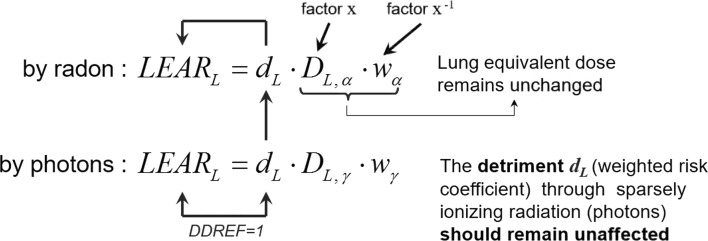
Dose and dose rate effectiveness factor DDREF is applied to the lung detriment *d*_L_ and refers solely to low-LET radiation (photons). For high-LET radiation (alpha radiation), no DDREF is applied, or DDREF = 1, respectively. However, an alteration of DDREF, nevertheless, would equally change the lung detriment due to radon exposure, too, since d_L_ is identical for any exposure. The other way around, an alteration of the lung detriment due to radon would equally change the risk of low-LET radiation. Thus, in order to avoid conflicts, the lung detriment *d*_L_ should remain unaffected by any revision of radon dose conversion. A revision of DDREF would affect in the same way both low-LET and high-LET radiation

There are some crucial implications of the alternative approach. The lung detriment d_L_, i.e. the weighted nominal risk coefficient for lung, in the first instance is determined for low-LET radiation, predominately based on the studies of the atomic bomb survivors (Life Span Studies, LSS) with respect to gamma radiation. The detriment *d*_L_ includes the DDREF and, thus, is altered if the DDREF is changed. It is important to state that the detriment *d*_L_ for low-LET radiation is identical to the *d*_L_ for high-LET radiation, e.g. alpha radiation due to radon exposure. Thus, in order to avoid conflicts the lung detriment *d*_L_ should remain unaffected by any radon dose conversion (Fig. 4). An assumed increase in the estimate of the risk of lung cancer is solely via the detriment. In the case of altering DDREF, with the dosimetric approach of ICRP in turn both are changed, the lung detriment and the lung equivalent dose. In contrast, with the alternative approach introduced here, the lung equivalent dose remains unchanged. If nevertheless dosimetric aspects due to the dosimetric approach give rise to an increased lung absorbed dose, the radiation weighting factor for alpha radiation might be put into consideration.

Radon and its progeny contribute via alpha decay to the lung absorbed dose. The ICRP dosimetric approach could lead to a change in the radon dose coefficients with respect to absorbed dose. However, the lung equivalent dose and effective dose remain unchanged. The DDREF refers to photon and electron radiation. It does not consider exposure due to alpha radiation. Radiation weighting factor may or may not remain unchanged. The nominal risk coefficients (relation between effective dose and damage) are adjusted by DDREF just only for low-LET radiation. This adjustment does not affect the estimated risk of radon exposure.

### Discussion and conclusion

There seem to be several unsolved conflicts with the latest radon-related ICRP recommendations. The problems refer not only to the quantitative changes in the dose estimates per unit exposure, or, respectively, the dose coefficients (approximately factor 2 or more or less), but also predominately even discrepancies in the ICRP framework of radiation protection in general.

The approximately doubling of the dose coefficients would be of rather dramatic consequence. Doubling the doses from radon, which is the most relevant natural source of radiation at all, means that the entire population gets an annual dose (due to existing exposure situation) that exceeds the dose limit for the public (for planned exposure situations). There is a fundamental different impact of doubling the risk estimates or of doubling the dose estimates. It will be hard to communicate why in radiation protection we are in a variety of situations concerned with fractions of mSv, on the one hand, and casually offer a permanent dose that will come up with such an exorbitant higher value, on the other hand. Thus, we have to be extraordinarily careful with changes in dose coefficient, and if we change, we have to have an imperturbable firm database. At present, it can be judged for both the epidemiological approach and the dosimetric approach in this sense the database is not sufficiently resilient.

The alternative approach presented here may avoid some of the shortcomings with respect to both principal conceptual aspects and practical implications. The detriment-adjusted risk of lung cancer per unit lung equivalent dose is identical for all radiation qualities and should be unaffected by new radon risk estimates. Actually, the doubling of the latter may be represented by setting DDREF = 1, while keeping the dose coefficient unchanged. In consequence, this procedure would avoid much of the inconsistencies and problems that we would be faced with if radon doses would be changed.

The average annual effective dose for natural radon remains ca. 1 mSv. Nevertheless, the new risk estimations of ICRP 115 (2010) will be taken into account. The DDREF must not be different for various radiation qualities. The existing dose system remains unchanged.

## Overall discussion

The four presentations described and summarised above led to a controversial and vivid discussion. This discussion is reported below based on the notes taken by WU Müller (chair of discussion session), A Giussani and W Rühm (rapporteurs).

Initially, some general issues were discussed such as, for example, the fact that ICRP proposes radon to be treated as an existing exposure situation. This was questioned for the case where building materials release radon and expose the inhabitants, a scenario which could be seen as a planned exposure situation. Because the source of radiation cannot be fully controlled in this case, however, ICRP has decided to treat this as an exposure from naturally occurring radioactive material (NORM) which is considered as an existing exposure situation. Afterwards, more specific questions with regard to radon were addressed.

### Are the radon dose coefficients in ICRP 115 (2010) reasonable?

An answer of this question was considered difficult, because calculation of new radon dose coefficients as those presented in ICRP 115 (2010) requires many assumptions and involves considerable uncertainties. For example, in the ICRP human respiratory tract model, the target cells are supposed to lie in a depth of 15 μm, although it is still unclear what exactly the target cells in the lung are and where exactly they are located. The biokinetic models used by ICRP are also subject to uncertainties, as is the value for the relative biological effectiveness for alpha particles used to calculate radon dose coefficients. In this context, it is important to recall that these sources of uncertainties are present for all radionuclides for which the dosimetric approach is applied and that the dose coefficients proposed by ICRP are meant as reference values to be used as a tool to control occupational exposures to ionising radiation.

All participants agreed that it is amazing how well the dosimetric and the epidemiological data agree, given the conceptual differences in the two approaches and the uncertainties involved. Indeed, the change in dose conversion factors by a factor of about two as proposed by ICRP should be seen in the light of the existing uncertainties. In order to improve the situation, efforts are necessary to identify and reduce those uncertainties. The complexity of the system allows for much tuning and also misuse. Quantifying the uncertainties involved with these parameters might help explaining the way the calculations were made and also explaining any decision of change. To reduce uncertainties involved in that procedure is not the basic task of ICRP, but it was suggested including this in the list of ICRP research topics that is currently under preparation, to improve the current system of radiological protection.

Uncertainties involved in the epidemiological approach include the fact that current ICRP publications do not yet adequately use the data obtained in the largest uranium miner study, the Wismut study in Germany. Recent evidence from this cohort based on data from miners hired after 1960, i.e. a population for which radon exposures were rather low, suggests values for the excess relative risk per WLM that are about a factor 2 lower than those assessed in the French and Czech miners and used by ICRP [thus, the Wismut data are closer to the values of ICRP 65 (1993)]. A future pooling of the Wismut study with the other major studies in the field is planned but will probably not provide results before the next 2–5 years. Such pooling efforts may help to test the epidemiological data for heterogeneity by leaving out one study after the other and comparing the pooled results of the remaining studies. Nevertheless, it was also noted that radon and radon progeny are among the very few examples where quantitative risk evaluations on exposed human populations exist.

With publication 115 and the upcoming reports on Occupational Intake of Radionuclides (OIR Report Series), ICRP proposes that radon should be treated for the first time like all other radionuclides after incorporation, but without ignoring the available epidemiological data. It was clear to the workshop participants that ICRP 115 (2010) is already published and that ICRP will recommend the use of the dosimetric approach to convert WLM to effective dose and a numerical value of 12 mSv per WLM. A clear advantage of the dosimetric approach is that it can provide radon doses to all target organs.

### What are the consequences of ICRP 126 (2014) for practical radiological protection?

A major conceptual topic raised in the discussion related to the fact that no difference is made in the report between smokers and non-smokers, although it has been demonstrated scientifically in epidemiological studies that the absolute radon risk of smokers is much higher than that of non-smokers. It might well be that this concept results in an over-protection of some and an under-protection of others. It was also emphasised, however, that ICRP recommendations are meant for a mixed population and that the concept of effective dose is defined for a reference individual for regulatory and surveillance purposes. The approach proposed in ICRP 126 (2014) is an integrated and simplified approach that does not, for example, require differentiating between homes and workplaces. Differentiating between smokers and non-smokers may open the room for other individual aspects such as differences in radiosensitivity between males and females, differences in lifestyle that influence radiosensitivity or genetic predisposition, which in turn may impose major ethical and legal problems if included in the system of radiological protection. Other aspects related to this problem include the fact that smokers can change their habits to reduce their risk to radon, while ex-smokers, who are also subject to an increased radon risk, cannot. As a solution to these problems, it was suggested regulating radon exposure only for never smokers, while making smokers and ex-smokers aware that their lifestyle is increasing their radon risk beyond that of a never smoker. This issue was discussed without a clear conclusion.

In terms of putting ICRP 126 (2014) into practice, a clear answer to the above-mentioned question still requires further analyses of the exposure scenarios and radon concentrations typical for a certain country. It is probable, however, that management of radon concentrations and radon exposures would be sufficient in most cases, at least for Germany, and there will be only few cases where the dose conversion convention will be needed.

### Does the system of radiological protection tolerate frequent changes?

It was emphasised that the basic principles characterising the ICRP system of radiological protection did not change much since they were introduced in 1977, and changes proposed in any numerical values, for example, of dose conversion coefficients showed only minor consequences. Moreover, those changes that did take place were always proposed on solid scientific grounds. For example, when the tissue weighting factors and, to a lesser extent, the radiation weighting factors were changed from ICRP 26 (1977) to ICRP 60 (1991) and then again from ICRP 60 (1991) to ICRP 103 (2007), it was because new epidemiological evidence for incidence had become available. Similarly, the change from the annual limit for effective dose of 50–20 mSv was proposed, when more reliable data from the atomic bomb survivors had become available. It was agreed that good scientific arguments are a prerequisite for any proposed changes to be accepted by the affected population, either the public or workers. If good evidence is not available, any change is difficult to justify and to communicate. It was noted that if changes are implemented, they should not occur too often, to avoid the impression that the system of radiological protection is unstable.

It is also important to note that although ICRP 115 (2010) focuses on radon, the changes proposed in ICRP 103 (2007) for the values of the radiation and tissue weighting factors and for the definition of the effective dose as the weighted sum of sex-averaged equivalent doses to the target tissues will result in changes in dose coefficients for most other radionuclides [ICRP 130 (2015)]. Changes to biokinetic and dosimetric models will also result in changes in dose coefficients for the inhalation and ingestion of radioisotopes of other elements.

Finally, it was observed that changes in ICRP recommendations may not be so critical per se, but there will be problems if these changes are not implemented internationally in a harmonised way. This problem was exemplified by the fact that Switzerland is currently the only country which implemented the changes proposed in ICRP 115 (2010), resulting in an “official” increase by a factor of 2 of the annual effective dose of the population, although the exposure conditions were the same as before. This was not done in the neighbouring countries.

### How should scientific uncertainties govern practical guidelines in radiological protection?

The participants agreed that the issue of uncertainties is important not only for radon. In general, the uncertainties involved in internal dosimetry are large, although in some cases, such as for radon, the scientific knowledge on the health effects resulting from radiation exposure is advanced, due to the existence of epidemiological studies on exposed human cohorts. In the light of the uncertainties, expert judgement is needed before recommendations can be given. By definition, the resulting numerical values used in operational radiation protection are without uncertainties. For example, in ICRP 92 (2003) a large range of different RBE values are reported for alpha radiation. Despite this large range, a single numerical value of 20 has then been recommended to be used as the weighting factor *w*_R_ for alpha radiation for the purpose of radiation protection without any attempt to quantify any uncertainty associated with this value. Based on this recommendation, radiation protection is then performed by applying the optimisation principle of keeping the exposure as low as reasonably achievable.

One should keep in mind that radiation protection should be seen to provide simple tools to be applied effectively for regulatory purposes only, with no claim of absolute scientific accuracy. In any case, although the uncertainties are rather high, radiation protection is frequently in a better position with respect to risk assessment than is the case for chemical or other types of noxious exposures.

For the specific problem of dose conversion factors for radon addressed in the present workshop, it became obvious that the available scientific evidence for the dose conversion factor does not allow a clear distinction between the values of 5 and 12 mSv WLM^−1^. This is because more recent data on RBE for alpha particles from the Mayak cohort may question the results of the dosimetric approach, while more recent data from the Wismut study may question the current epidemiological estimates. Others came to the conclusion that given the large uncertainties involved there is no compelling scientific evidence for any change (i.e. the values can be considered not to be significantly different if the related uncertainties are considered).

For the specific example of radon and radon progeny as alpha emitters, it was stressed that at least one source of uncertainty is comparably small: there are good scientific reasons that the numerical value of the dose and dose rate effectiveness factor (DDREF) as proposed by ICRP for alpha radiation is one indeed, because the DDREF is related to DNA repair which is low for alphas. One should also keep in mind, however, that for alpha radiation, a single cell may get a much higher dose than the average organ dose would suggest.

Finally, it was mentioned that physical dose measurements also include uncertainties and that ICRU is just finishing a report on radon measurements, which will include guidance on how to measure and report radon exposures (ICRU Report 88: Measurement and Reporting of Radon Exposures; to be published).

### Does the concept of effective dose work for radon exposures?

In general, it was emphasised that effective dose was introduced for planning purposes and for optimisation of radiation exposures at working places as a tool for implementing the radiation protection principles. It allows for a combination of various exposure scenarios (external, internal), and comparing the result with dose limits which are also given in terms of effective dose.

Whereas effective dose is useful and extremely practical for the purpose of regulation and control, it is, however, not a direct measure for radiation risk but just a risk-related quantity, and that it should not be used to calculate risk. The opinion was expressed that effective dose is particularly useful to compare the magnitude of risk in exposure scenarios where the risk is not known. For radon, however, effective dose is not needed because the risk from radon exposure is already known. Additionally, effective dose should be applied with care when local exposures are dominant (e.g. when only one exposed organ is involved). In that case, use of organ doses is more appropriate. In the case of radon, it makes little sense to weight doses over all target regions of the body, because dose is given overwhelmingly to only one target tissue, i.e. the lung (although the equivalent dose to the lung is also defined as the weighted average of the doses to different subregions of the lung).

In this context, it was stressed that the physical dose (given as absorbed dose in Gy) is not at all affected by the considerations on dose conversion factors for radon to obtain effective dose (in Sv). The latter contains a lot of judgement that may change with time, whereas the physical dose remains the same. The issue, organ absorbed dose vs effective dose, can cause serious problems in communication with the public. This was the case, for instance, after the Fukushima accident, when in the beginning dose was communicated as thyroid dose, while it was later communicated as effective dose creating confusion and scepticism in the population.

As was already indicated above, the approach formulated in ICRP 126 (2014) means that effective dose is only needed in very few cases to protect against radon exposures.

### How should any changes in the pie chart of exposures from natural and man-made sources of ionising radiation be communicated?

The question was raised whether the pie charts which are used to compare occupational and public exposures in terms of annual effective dose are at all useful, and whether they have not been used beyond the original purpose. It should be kept in mind that effective dose was developed for practical radiation protection purposes only, and not for risk communication to the public. Unfortunately, the concept of effective dose has been misused for several years, so it is difficult now to go back although it is evident that its use is often not scientifically correct and goes beyond the original intended meaning.

Alternatives are, however, difficult to find, and it was proposed to, at least, show two pie charts, one for occupational and one for public annual exposure. For example, presentation of a pie chart on occupational exposures and a second one on medical exposure, which was suggested to be called “patient exposure”, would facilitate to communicate the specific characteristics associated with these two exposure scenarios such as, for example, the fact that for patient exposures, a clear medical benefit from the applied diagnostic procedure should be obvious for the individual patient.

Another fundamental issue with the pie chart is that it presents average values for the whole population resulting in very low values, for example, for exposures resulting from the use of nuclear power.

On the other hand, it was agreed that just providing numbers characterising the exposures of the various sources of ionising radiation is difficult to communicate to the public. This view was supported by saying that long and detailed scientific descriptions might be more difficult to be understood by the people, and simple tools such as the pie chart, if handled with care, might help to explain complex situations. An alternative approach could be to go for a column presentation of the various sources of exposures. This approach has already been used recently in the BfS report to the German Parliament on exposure of the population to ionising radiation (https://www.bfs.de/SharedDocs/Downloads/BfS/DE/fachinfo/parlamentsberichte-dip.html). It was noted, however, that some more explanation would be useful in that specific case. An advantage of the column presentation could also be that uncertainties can easily be included by adding uncertainty bars to each column representing exposure to each of the sources of ionising radiation. However, this may also increase the difficulty of the public to understand the overall issue.

In order to improve the situation, it was suggested that efforts should be made to get feedback from the population on the way how radiation exposures should be presented. It may also be of help if international and national bodies dealing with radiation protection such as ICRP or SSK could give advice. It was noted that recent activities of ICRP do include efforts to approach the general populations and the scientific community, such as public consultations on draft ICRP reports, dialogue actions with the population of Fukushima, or workshops on ongoing ICRP actions. Some participants of this workshop expressed the opinion that at least guidance on risk communication would not be the primary task of international bodies giving advice in radiation protection issues, and that this task should rather be considered by other international bodies or national governments.

## Overall conclusion

- It is evident that from time to time, changes in the current system of radiological protection and the dose coefficients included are needed. Such changes require, however, sound scientific evidence, which should be communicated to the regulators in a clear and understandable language.
- Related to that, the proposed changes should not be issued too quickly, to assure that the most recent scientific findings can be included in the analysis. This was identified as one of the problems with the recent update of dose conversion factors for radon which were published without considering the upcoming results from the German Wismut study. Publication of comprehensive ICRP reports after stakeholder involvement is preferred instead of first issuing basic messages through ICRP statements (as was done for radon and the lens of the eye).
- Implementation of ICRP recommendations is the task of regulators. It was concluded that regulators should take more time in analysing the consequences of any changes for practical radiological protection as proposed by ICRP. If there are good reasons, it is not compulsory for authorities to follow all ICRP recommendations, but deviations from ICRP recommendations by a regulator can be considered only if very well justified.
- Finally, it was felt that workshops with direct participation of ICRP representatives, as it was done for the present workshop, are very helpful.
